# Reproduction, abundance and survivorship of two *Alveopora* spp. in the mesophotic reefs of Eilat, Red Sea

**DOI:** 10.1038/srep20964

**Published:** 2016-02-10

**Authors:** Lee Eyal-Shaham, Gal Eyal, Raz Tamir, Yossi Loya

**Affiliations:** 1 Tel Aviv University, P.O. Box 39040, Tel Aviv 6997801, Israel; 2 The Interuniversity Institute for Marine Sciences, P.O. Box 469, Eilat 8810369, Israel; 3 Bar-Ilan University, Ramat-Gan 5290002, Israel

## Abstract

Although the study of coral reproduction has advanced tremendously over the last few decades, a particular gap exists in our knowledge of the reproductive modes of corals from ‘mesophotic coral ecosystems’ (MCEs) found at 30–150 m depth. Here, we report for the first time on the reproductive patterns, living cover, and survivorship under different light treatments of two scleractinian species from the MCEs of Eilat, Red-Sea: *Alveopora allingi* and *A. ocellata*. Both species are found exclusively within MCEs and are high in both abundance and relative cover. These species display a synchronous gametogenic cycles with consecutive oocyte growth and development. Peak of reproductive activity occurs in late summer (September-October), typified by accelerated oocyte growth, coinciding with the rise in seawater temperature. Estimates of fecundity show mean monthly maxima of 48.5 ± 26.3 and 23.5 ± 11.8 (Mean ± SE) oocytes per cm^2^ for *A. allingi* and *A. ocellata* respectively, prior to spawning. A comparison of light and temperature regimes in the shallow vs. MCE environments is presented, and the response of these species to changes in these parameters is discussed. A call encouraging the much-needed studies on the sexuality and reproductive modes of MCE coral species is expressed.

Sexual reproduction is a critical life-history function important in the maintenance and evolution of coral communities^1^. Presumably, coral species have adapted and evolved in response to physical (e.g. temperature, salinity, light intensity, sedimentation, etc.) and biological (e.g. competition, predation, diseases, etc.) factors, and their life-history strategies are expected to reflect the selection of traits adaptive to the various situations. Reproductive traits of scleractinians: coral sexuality (hermaphroditism vs. gonochorism), mode of reproduction (brooding vs. broadcast spawning), and timing of reproduction (seasonality, periodicity, and synchrony), vary among species^2^,^3^ and can have an important influence on the recovery of disturbed coral reefs^4^. Some of these reproductive characteristics (e.g. coral sexuality) were demonstrated to be highly conserved within groups and thus, indicate a strong systematic pattern in coral reproduction (reviewed by Baird *et al.*^5^). Such reproductive differences among species may derive from the particular habitat conditions in different zoogeographic regions^2^ and may also be induced by distinct environmental cues in proximate geographical habitats^6^. Although the study of coral reproduction has advanced tremendously over the last four decades, we are yet to understand fully the selective mechanisms and adaptive benefits that have led to this group’s reproductive strategies^5^,^7^,^8^,^9^. A particular gap exists in our knowledge of the reproductive modes of coral species from the ‘mesophotic coral ecosystems’ (MCEs), situated at depth of 30–150 m. MCEs are coral-dominated communities that occur in the deepest part of the photic zone^10^,^11^,^12^. The array of biotic and abiotic environmental variables prevailing in the MCEs vastly differs from those in shallow reefs and can potentially alter the life strategies of its resident species, particularly of sessile organisms such as scleractinian corals. The environmental circumstances which expose these reefs to different physical conditions than those in shallower reefs (e.g., limited light penetration, cool upwelling conditions, negligible wave effect, higher concentration of nutrients, etc.)^13^,^14^,^15^,^16^, are likely to have an impact on the reproduction of corals in this area. These environmental disparities have also given rise to the hypothesis that these reefs function as deep-reef refugia (DRRH) from various environmental disturbances, such as climate change^17^ and anthropogenic disturbances^15^,^16^. At present, due to their secluded existence (below recreational SCUBA diving depth), these mesophotic reef communities have remained relatively less studied^18^.

The Gulf of Aqaba/Eilat (hereafter referred to as GOA/E) is a desert-bordered, semi-enclosed gulf located at the northern tip of the Red Sea. This area is a well-studied marine system of great environmental importance: the coral reefs are situated at the northern limits of global coral-reef distribution and embrace a rich diversity of habitats, including shallow coastal lagoons, sea grass beds, mangrove stands, and coral reefs^19^,^20^. While the shallow reefs have been studied extensively during the past few decades^19^,^21^,^22^,^23^ the MCEs, even though well-developed along the GOA/E coastline to a depth of at least 130 m^24^, have been rarely approached, with very few articles describing MCEs of the GOA/E having been published in recent years, and none with respect to coral reproduction.

The small acroporid corals *Alveopora allingi* Hoffmeister, 1925 and *Alveopora ocellata* Wells, 1954 are among the most abundant coral species in the MCEs of the western coast of the GOA/E^24^. Here, we report for the first time on the reproductive biology (i.e., sexual system, gametogenic cycles, gamete development and fecundity), living cover and survivorship under different light treatments of these species from the MCEs of Eilat, Red Sea.

## Results

### *Alveopora allingi* and *A. ocellata*: sexuality, mode of reproduction, and criteria for developmental stages

**Figure 1:**
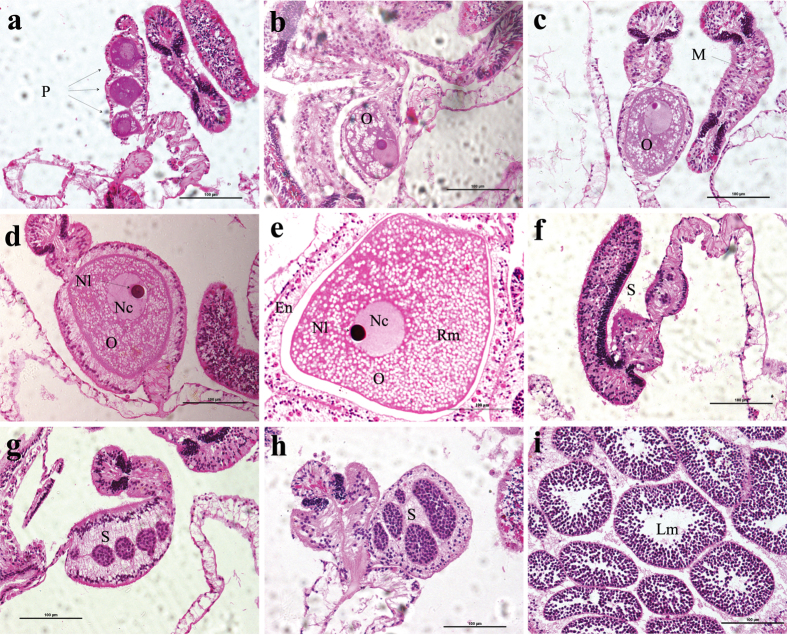
Photomicrographs of representative gametogenic stages typifying the corals *Alveopora allingi* and *Alveopora ocellata*. (**a**) primordial oocytes (i.e. early oogonia stage) (**b**) Stage І oocyte with enlarged interstitial cells (**c**) Stage ІІ oocyte enveloped by mesoglea (**d**) Stage ІІІ oocyte enveloped by mesoglea (**e**) Mature oocyte of stage ІV with well-distinguished vitelline membrane (**f**) Stage І spermaries constitute a small cluster of spermatocytes (**g**) Stage ІІ spermaries surrounded by mesoglea (**h**) Stage ІІІ spermaries showing early development of lumen (**i**) Stage ІV spermaries with spermatocyte tails in lumen. O: oocyte; S: spermaries Nl: nucleolus; Nc: nucleus; M: mesoglea; Rm: reservoir materials; En: endoderm; Lm: lumen; Vm: vitelline membrane; P: primordial oocyte. All scale bars 100 μm.

From the histological study it is evident that *A. allingi* and *A. ocellata* are hermaphroditic spawners, with each polyp having both male and female functions. Polyps of both species have 12 mesenteries per polyp. Oocytes and spermaries develop on separate mesenteries within each polyp (Fig. 1). The criteria for classification of gametocytes into developmental stages for *A. allingi* and *A. ocellata* were as follows: four developmental stages for oocytes and four for spermaries were defined based on Szmant-Froelich *et al.*^25^,^26^ and Glynn *et al.*^27^ (Supplement Table 1). The primordial oocyte (i.e. early oogonia stage) could hardly be distinguished from the interstitial cells and was rarely seen (Fig. 1a). When encountered, it was quantified and added to the sum of stage I. The other, more progressed, oocyte stages were easier to distinguish due to their relatively large size, well-defined boundaries and typical pinkish cytoplasm (Fig. 1). Throughout the breeding season, a considerable accumulation of cytoplasm in the oocytes was observed in the histological cross-sections (Fig. 1a–e). The spermatogonia were always grouped in clusters surrounded by a thin mesogleal layer, making them easier to distinguish. As the spermaries developed, the number of spermatocytes increased (Fig. 1f–i). At later stages (ІІІ) a central lumen started to develop (Fig. 1h). Sperm tails were observed only in the final stage (ІV) (Fig. 1i). Throughout the reproductive season the spermaries were not very common in the polyps. Only at the very end of the season, just before spawning, were the spermaries seen to be equally spread within the polyp, as were the oocytes. No planulae larvae were observed in any sample.

### *A. allingi*: gametogenesis and fecundity

**Figure 2:**
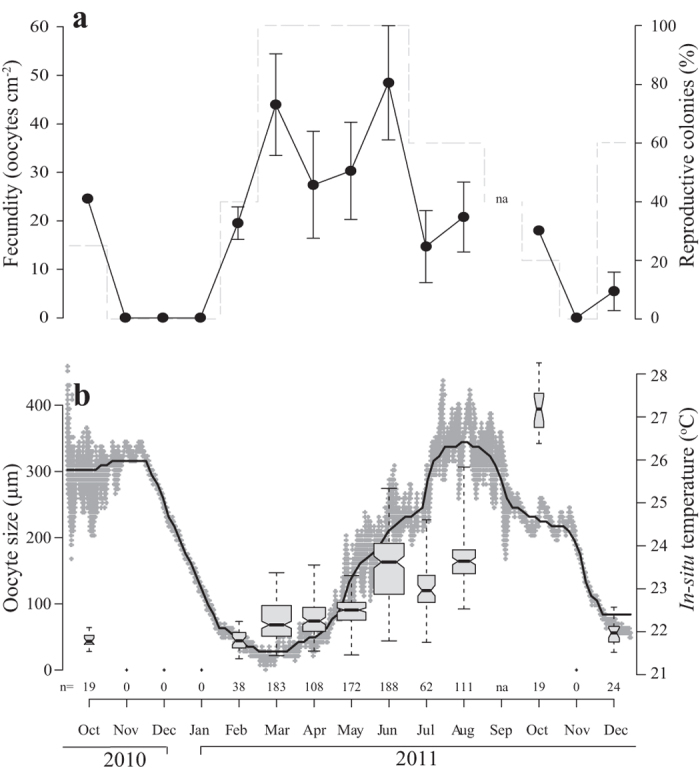
Reproductive parameters of *Alveopora allingi*. (**a**) Fecundity values and percentage of reproductive colonies. Black dots: monthly average fecundity values (number oocytes per cm^2^ of coral tissue); Error bars are ± SE; n = 3–5 colonies. Dashed line: percentage of reproductive colonies. (**b**) Temporal pattern of average oocyte size (μm) with chronological temperature data measured from October 2010 through December 2011. Gray boxes represent monthly average oocyte size measurements; center black lines show the medians; box limits indicate the 25th and 75th percentiles; notches are defined as +/−1.58*IQR/sqrt(n) and represent the 95% confidence interval for each median; whiskers extend to minimum and maximum values; width of the boxes is proportional to the square root of the sample size; n = 0–188 individual oocytes measurements as indicated under the boxes. Gray plus sign represent *in-situ* ten minute interval temperature measurements at 50 m depth (°C); black line represents running median of 100 measurements which eliminates the daily variation in temperature.

The reproductive cycle of *A. allingi* is annual, with oogenesis development of about eight months; oocytes first appear in February, maturing in late August to early September and reaching a diameter of *ca.* 390 μm (Fig. 2). One exception to this scheme was a colony that contained ripe gametes in October. Up to four oocytes of stages І-ІІІ, mostly at the same reproductive stage, were seen in a mesentery and up to three oocytes from stage ІV. Male gonads, which appear as clusters of male germ cells, were first observed in May in the mesenterial endoderm and matured approximately four months later. Up to two developmental stages appeared adjacent in the same mesentery.

Oocytes which developed to maturity increased in size from an average of 45.7 ± 15.0 μm (mean ± SD, *n* = 38) in February to an average of 166.0 ± 34.1 μm (mean ± SD, *n* = 111) in August, and an average of 393.4 ± 33.6 μm (mean ± SD, *n* = 19) in October. In October, when the percentage of reproductive colonies was only 20%, the average oocyte size was largest (Fig. 2). Data from September 2011 are absent; however, data from September 2012 revealed that all sampled colonies of *A. allingi* (n = 5) were devoid of gonads.

**Figure 3:**
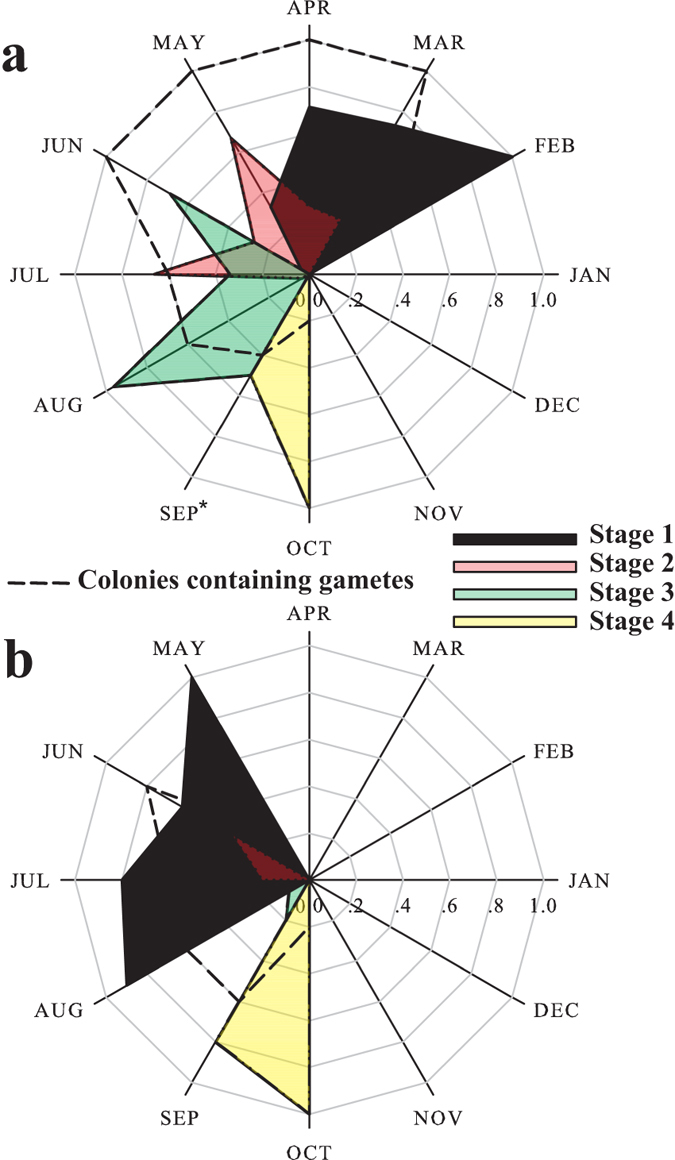
Summary of oogenic and spermatogenic annual patterns in *Alveopora allingi* determined by examination of histological cross-sections; stages as in Supplement Table 1. (**a**) Monthly percentage of oocytes quantified as one of the four different developmental stages. Dashed line: Monthly percentage of colonies containing oocytes (**b**) Monthly percentage of spermaries quantified as one of the four different developmental stages. Dashed line: Monthly percentage of colonies containing spermaries. Asterisk (*) represents estimation of monthly percentage of colonies containing oocytes.

In the sampled colonies primordial oocytes and stage І oocytes were observed from February (100%) to June (4%). Stage І oocytes had an average size of 52.5 ± 13.2 μm (mean ± SD, *n* = 20). Stage ІІ oocytes first appeared in March, peaking in percentage in May and July at 67% and 66%, respectively, and declining in August to 4%. Mean diameter of stage ІІ was 96.0 ± 19.8 μm (mean ± SD, *n* = 20). Stage ІІІ oocytes prevailed from June with 68%, through August with 96%, with an average diameter of 181.5 ± 46.7 μm (mean ± SD, *n* = 20). Stage IV oocytes were detected for the first time in October 2011 with an average size of 393.4 ± 33.5 μm (mean ± SD, *n* = 19) (Figs 2 and 3a). However, in September 2012 none of the sampled colonies contained gametes and, therefore, were assumed to have reached at the post-spawning stage. This led us to the assessment that one year earlier, in September 2011, approximately 50% of Stage IV oocytes had been present. Stage І spermaries were formed in May and were present till August. The highest percentage (100%) of stage І was observed in May. Stage ІІ spermaries were observed in June and July only. Stage ІІІ was evident only in August, but the data indicate that they were also present in September. Stage ІV was observed in October but was inferred to have been present also in September (Fig. 3b).

During the reproductive period the highest estimated fecundity value of 48.5 ± 26.3 oocytes per cm^2^ of coral tissue (mean ± SE, *n* = 5) was documented in June 2011, while the lowest estimated fecundity value of 5.45 ± 6.8 oocytes per cm^2^ of coral tissue (mean ± SE, *n* = 3) was documented in December 2011 (Fig. 2a).

### *A. ocellata*: gametogenesis and fecundity

**Figure 4:**
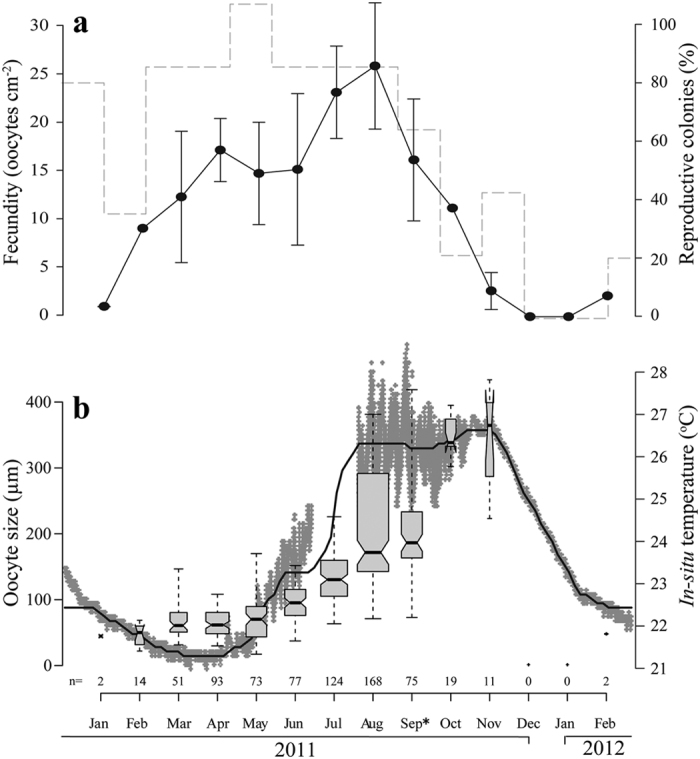
Reproductive parameters of *Alveopora ocellata*. (**a**) Fecundity values and percentage of reproductive colonies. Black dots: monthly average fecundity values (number of oocytes per cm^2^ of coral tissue); Error bars are ± SE; n = 2–5 colonies. Dashed line: percentage of reproductive colonies. (**b**) Temporal pattern of average oocyte size (μm) with chronological temperature data measured from January 2011 through February 2012. Gray boxes represent monthly average oocyte size measurements; center black lines show the medians (see Fig. 2. for explanations of all other signs). Asterisk (*) represents data from September 2012.

**Figure 5:**
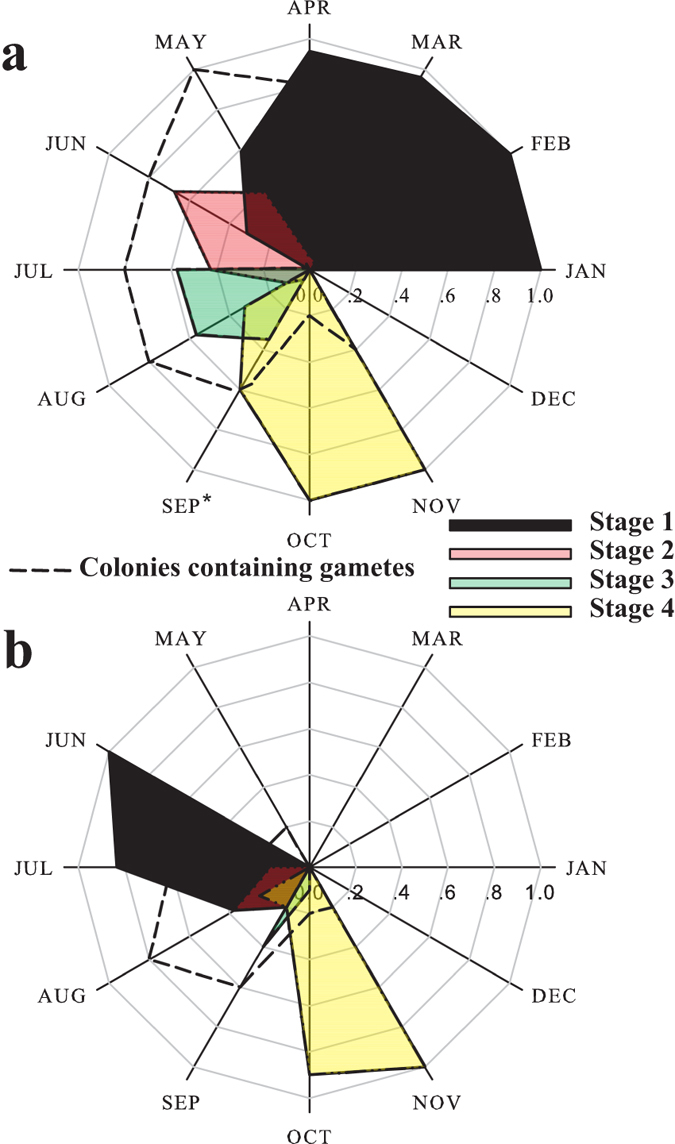
Summary of oogenic and spermatogenic annual patterns in *Alveopora ocellata* determined by examination of histological cross-sections; stages as in Supplement Table 1. (**a**) Monthly percentage of oocytes quantified as one of the four different developmental stages. Dashed line: Monthly percentage of colonies containing oocytes (**b**) Monthly percentage of spermaries quantified as one of the four different developmental stages. Dashed line: monthly percentage of colonies containing spermaries. Asterisk (*) represents data from September 2012 of monthly percentage of colonies containing oocytes.

The reproductive cycle of *A. ocellata* is annual, with an oogenesis development of about 10 months; oocytes appear in January and maturation takes place from late September to early October, with a delayed gamete release by some colonies in November. The oocytes reached *ca.* 350 μm in diameter (Fig. 4). There were up to four oocytes in a mesentery, mostly at the same developmental stage. The spermaries were first observed in June. Development continued until late September/beginning of October and in a few cases until November (Fig. 5). Up to two developmental stages appeared adjacent in the same mesentery.

Oocytes which developed to maturity increased in size from an average of 46.7 ± 14.1 μm (mean ± SD, *n* = 16) in January and February to an average of 353.7 ± 28.3 μm (mean ± SD, *n* = 19) in October (Fig. 4b). The majority of the colonies spawned between the end of September and the beginning of October. However, 40% of the sampled colonies still contained large and mature gametes in November (Figs 4a and 5a). Data from September 2011 are lacking; but those from September 2012 indicate that 60% of the sampled colonies of *A. ocellata* contained mature oocytes and spermaries, and hence were yet to release their gametes into the water column.

In the sampled colonies primordial oocytes and stage І oocytes were observed from January (100%) to June (31%). Stage І oocytes had an average size of 52.3 ± 9.1 μm (mean ± SD, *n* = 20). Stage ІІ oocytes appeared in minute quantities in March and April (4% and 5%, respectively) and rose to 68% in June. Average diameter of stage ІІ was 105.8 ± 22.6 μm (mean ± SD, *n* = 20). Stage ІІІ oocytes were hardly seen in May and June, at 3% and 1%, respectively, and peaked in July and August to 57% in both. The average diameter of stage ІІІ was 167.8 ± 30.2 μm (mean ± SD, *n* = 20). The fourth stage of oocytes was seen for the first time in August 2011 (32%) and dominated in October and November, with 100% of the oocytes in stage ІV. In September 2011 60% of the oocytes were at stage ІV (Fig. 5a). The average oocyte size was 319.5 ± 28 μm (mean ± SD, *n* = 20) (Fig. 4b). Stage І spermaries were observed from June (100%) through August and were inferred to be present till September. Stage ІІ spermaries were observed from July (17%), peaked in August (37%), and were inferred to be present also in September. Stage ІІІ was not evident in this species but the data suggest that they may be present in September and October. Stage ІV in this species was observed from August till November (Fig. 5b).

During the reproductive period the highest estimated fecundity value of 23.5 ± 11.8 oocytes per cm^2^ of coral tissue (mean ± SE, *n* = 4) was documented in August, while the lowest estimated fecundity value of 0.9 oocytes per cm^2^ of coral tissue) (*n* = 2) was documented in January (Fig. 4a).

### Water temperature vs. oocyte size

There is a significant correlation between seawater temperature (at 50 m depth) and average oocyte size in both *A. allingi* (Pearson Correlation, R = 0.418, p < 0.001) and *A. ocellata* (Pearson Correlation, R = 0.569, p < 0.001). The most developed oocytes appeared in conjunction with the highest seawater temperature (Figs 2b and 4b).

Temperature measurements show smaller diurnal and seasonal temperature variation in the MCEs than in the shallow reef (Supplement Fig. 1). Annual lowest and highest temperature values were measured in the shallow reef.

### Living cover: ecological photo survey

**Figure 6:**
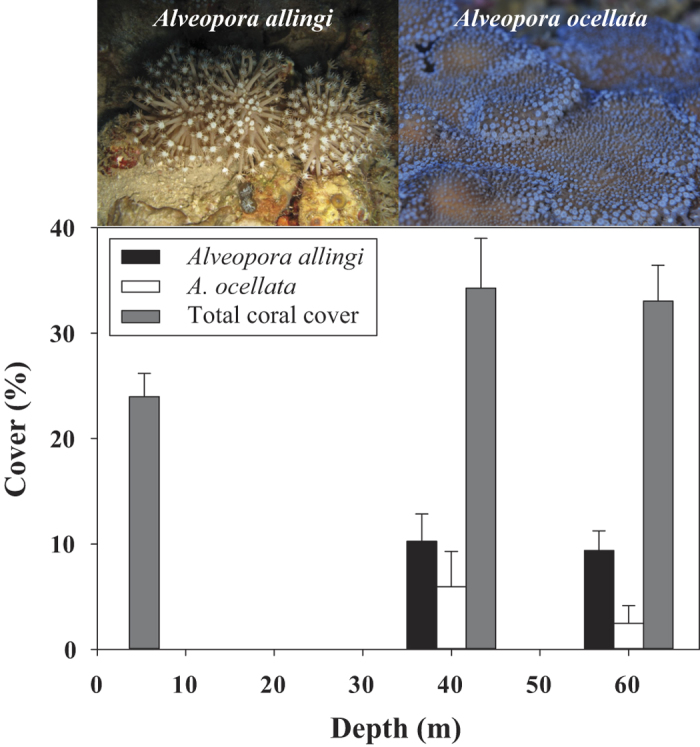
Living coral cover along a depth gradient (2, 40 & 60 m) off the Interuniversity Institute (IUI), Eilat. Gray bars represent total coral cover, black bars represent *Alveopora allingi* and blank bars *A. ocellata* living cover within the same quadrats. n = 30 quadrats per depth. Error bars = 95% CI of the mean.

The ecological photo survey showed a significantly higher coral cover at mesophotic depths (i.e. 40, 60 m) than in the shallow reef (One-way ANOVA, F = 10.827, p < 0.001) (see Supplement Tables 2 and 3) with total corals cover of *ca.* 24%, 34%, and 33% at 2, 40,and 60 m, respectively. *A. allingi* and *A. ocellata,* which are absent from the shallow reef, constitute *ca.* 30% and *ca.* 17% of the total coral cover at 40 m depth and *ca.* 28% and *ca.* 8% of the total coral cover at 60 m depth, respectively (Fig. 6).

### Coral survivorship under controlled light regimes

**Figure 7:**
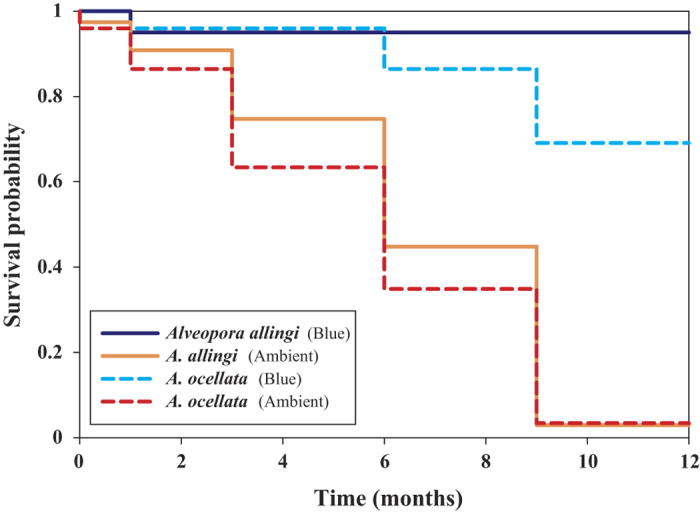
Kaplan-Meier survivorship probabilities of mesophotic *Alveopora allingi* (full line) and *A. ocellata* (broken line) under ambient light matching the light at 3 m depth and using blue-light filter (matching the light at 40 m depth), in running open-circuit seawater aquaria at the IUI during 12 months. Cold color curves (blue and cyan) represent the probability of colonies survival under blue light, equivalent to mesophotic depth, and warm color curves (orange and red) represent the probability of colony survival under shallow water light conditions. The colonies were collected at 60 m depth. n = 5, 15, 5, and 10, respectively (set-up in Supplement Fig. 3).

Survivorship probabilities monitored under different light conditions in the outdoor running seawater aquaria at the IUI of *A. allingi* (log-rank test, *Χ*^2^ = 13.758, p < 0.001) and *A. ocellata* (log-rank test, *Χ*^2^ = 11.788, p < 0.001) were significantly higher under blue-light conditions than under ambient light, during the 12 months of the experiment (Fig. 7). Mean (±SE) survival time under ambient light conditions for *A. allingi* and *A. ocellata* was 6.77 ± 0.87 and 6.77 ± 0.87, respectively. Under blue light conditions mean survival time of *A. allingi* and *A. ocellata* was 8.72 ± 0.68 and 7.93 ± 0.54, respectively.

### Downwelling irradiance measurements

Measurements of percentage of photosynthetically active radiation (PAR) from surface irradiance revealed higher light intensities and higher light variation in the shallow reef. During most of the year, the percentage of PAR varied from 20–100% of the surface irradiance in the shallow reef (0–20 m depth), while in the mesophotic reef (60 m depth) the percentage of PAR demonstrated a more constant trend, varying from a minimum of *ca.* 0.5% in March to a maximum of *ca.* 3% in August (Supplement Fig. 2).

## Discussion

This study is the first to document the reproductive cycle of corals in the MCEs of the northern Gulf of Aqaba/Eilat (GOA/E), Red Sea. The studied species, *Alveopora allingi* and *A. ocellata*, are hermaphroditic broadcasters which exhibit a synchronous gametogenic cycle with consecutive oocyte growth and development. Peak reproductive activity occurs in late summer, when the most rapid growth in the diameter of the oocytes takes place, coinciding with the rise in seawater temperature. The oogenic cycle of *A. allingi* is shorter than that of *A. ocellata*: oogenesis in *A. allingi* begins in February and is completed in eight months, while oogenesis in *A. ocellata* begins in January and extends over a period of 10 months. Spermatogenesis in both species occurs over a brief period of four months (Figs 3b and 5b). Oogenesis precedes spermatogenesis by four months in *A. allingi* and six months in *A. ocellata*. Throughout the study, no planulae were observed in any of the histological cross-sections of the sampled colonies; i.e. the possibility of brooding was ruled out.

Although spawning was not directly observed, histological evidence clearly indicated that *A. allingi* and *A. ocellata* are broadcast spawners. The main spawning event was thus assumed to have occurred in September 2011 for *A. allingi* and in October 2011 for *A. ocellata*. This conclusion is derived solely from the histological samples and relies on the interval between samples containing ripe gametes and samples devoid of gametes or possessing immature gametes. For both species, a second smaller episode of spawning may have occurred, one month following the main spawning event. Samples collected in October for *A. allingi* and in November for *A. ocellata* revealed a few exceptional colonies that still contained ripe gametes. Hence, their late spawning was inferred from the histological study. Multiple spawning events in corals are not unusual and have been previously reported in the literature^2^,^5^,^7^,^28^,^29^.

The close yet discrete timing in these two congeneric species may indicate potential temporal reproductive isolation in the MCEs, a phenomenon first described from coral populations in the shallow reefs of the GOA/E^28^. However, as environmental conditions in the MCEs differ from those in the shallow reefs^12^,^14^, the possible cues which stimulate this process need to be reassessed.

Fecundity has been observed to vary with depth, either positively (reviewed in Harrison and Wallace^7^) or inversely^30^. In the Caribbean, *Orbicella faveolata* fecundities were shown to be significantly higher in MCEs (35–40 m) than at shallow (5–10 m) sites, while no difference was found in *Porites astreoides* fecundity between mesophotic, mid-depth and shallow sites^31^. In the Red Sea lower fecundities were found in deeper (25–45 m) colonies of the brooder coral *Stylophora pistillata* than at shallow sites (5 m), but values varied considerably between successive years in shallow-water populations^32^. In the MCEs at Okinawa, Japan, fecundity of *Acropora tenella* was shown to be lower than that of most acroporids^33^. In this study, *A. allingi* and *A. ocellata,* which are absent from the shallow reef in the GOA/E, demonstrated monthly maximum estimated fecundity values of 48.5 ± 26.3 and 23.5 ± 11.8 oocytes per cm^2^ of coral tissue in the MCEs, respectively. The fact that a higher fecundity was observed three to four months prior to the spawning period of *A. allingi* and *A. ocellata* (Figs 2a and 4a), may imply a possible absorption of oocytes in the pre-spawning period.

Environmental factors can have diverse and often strong effects on reproduction^34^. However, the extent to which population reproductive traits respond to different environmental factors is not entirely clear. Variations in breeding methods due to environmental conditions have been studied in many higher plants^35^ and animals^36^,^37^. The mode of reproduction in corals demonstrates ecological features common to both plants and animals^38^, and challenges simple predictive models. It has been hypothesized to be related to various factors: e.g., colony size^2^, habitat^39^, morphology^40^, life- history strategy^38^,^41^, and depth^7^. Harrison and Wallace^7^ and Harrison^9^, have concluded after summarizing the known coral reproductive characteristics that there are general systematic trends in coral sexuality (hermaphroditism vs. gonochorism) but not in embryonic development (broadcasting vs. brooding). Further evidence of this has been provided through the use of new molecular phylogeny methods (reviewed by Baird *et al.*^5^). However, even this trait appears relatively stable, as only 13 of a total of 111 genera whose reproductive biology is known contain species that both brood and spawn, among them the genus *Alveopora*^5^: two species are hermaphroditic brooders and five are hermaphroditic spawners (Table 1). Interestingly, the coral species *A. allingi* and *A. ocellata* from the MCEs of the GOA/E demonstrate a different reproductive mode from their congeneric brooder *A. deadalea* which resides in substantial physical proximity, in the shallow reefs of the GOA/E^28^.

Environmental conditions in the MCEs vary quite widely from those of the shallow reef: e.g., lower temperature oscillations (Supplement Fig. 1), a lesser degree of solar irradiance (Supplement Fig. 2), and moderate currents and wave action^14^. Hence, these reefs are typified by relatively stable and benign environmental conditions and, furthermore, may suffer to a lesser degree from certain man-made disturbances (e.g. climate change, over-fishing, pollution, etc) which threaten shallow coral reefs^14^. MCEs therefore provide a potentially more favorable environment for those species that outcompete others in this particular environment. The two studied *Alveopora* species that thrive in these unique environmental conditions of the Eilat MCEs contribute a mean cover of 9.4% for *A. allingi* and 5.9% for *A. ocellata* from the total benthic cover at 60 m depth^24^,^42^. They are absent from the shallow reefs in Eilat (Fig. 6), probably due to stressful, excessive light conditions. Manipulative survival experiments revealed a low competence of their colonies to survive the light intensity and spectrum range typical of the shallow reefs in Eilat (Fig. 7). The reef flat in the GOA/E constitutes a physically-controlled coral community e.g., it is exposed to natural disturbances, such as periodic but unpredictable extreme low tides^22^,^41^ and severe southern storms^43^, which are known to cause severe damage to the scleractinian coral communities. Such communities are also negatively affected by disturbances attributed to human activities that greatly perturb and destabilize the shallow reefs: e.g., inexperienced divers’ activity^44^, sewage spillages and oil spills^22^,^45^, sedimentation, overfishing, and dredging^21^,^22^. The relatively greater variability and higher amplitude of those environmental parameters that characterize the shallow reefs of Eilat are much reduced at the MCEs of these reefs, as they are spatially more distant from shore and occur at greater depths (30–150 m) (see Supplement Figs 1 and 2).

Stimson^39^ described brooding as the predominant mode of reproduction in shallow reefs in Hawaii. He reported early sexual maturity in corals inhabiting shallow water, and hypothesized that there might be selection for species with a high reproduction rate in disturbed habitats. Similarly, Van Moorsel^46^ suggested that the reproductive traits of brooding corals could be related to the predictability of their habitat. He documented early reproduction in colonies of *Agaricia humilis* (colony size *ca.* 30 mm corresponding to an age of less than two years) from a habitat with a relatively low level of predictability, in contrast to later reproduction (colony size *ca.* 108 mm corresponding to an age of less than 4–5 years) in colonies of *Agaricia agaricites,* which are mainly confined to the more environmentally predictable reef zone. He suggested that energy allocation in fragile environments is channeled more rapidly to reproduction than in stable environments. This idea was extended by Szmant^2^ who suggested that brooding evolved in those coral species that occupy less stable habitats. Edinger and Risk^47^ consequently hypothesized that brooders have a greater capacity to recruit and survive in marginal conditions (e.g., *Alveopora japonica*^48^). Hence, the difference in reproductive mode between *A. allingi, A. ocellata,* and *A. daedalea*^49^ within such a close spatial range (150–250 m horizontally and 55 m vertically), may support the assumptions by Loya^50^, Szmant^2^, and Van Moorsel^46^ with respect to potential link between the relative predictability of the habitat and coral’s reproductive mode: while the hermaphroditic brooder *A. daedalea* inhabits the shallow reef of the GOA/E, an area exposed to recurrent disturbances, the hermaphroditic broadcast spawners *A. allingi* and *A. ocellata* thrive solely in the more stable habitat of the deep reef. Clearly, such a hypothesis should be approached with caution, and these observations do not exclude the possibility that other species, which are limited in their spatial distribution to the MCEs, may prove to be brooders. Nevertheless, it meant to highlight an important gap in our knowledge of MCEs, and encourage the much needed extensive studies on the sexuality and reproductive modes (*sensu* Baird *et al.*^5^) of MCE coral species. Furthermore, and specifically with respect to the current study, in light of the new phylogenetic affiliation of the genus *Alveopora*, which was transferred from the family Poritidae to the Acroporidae, and until a full phylogenetic evaluation of these species is completed^51^, such comparisons of reproductive traits should be approached with caution.

Corals demonstrate considerable physiological flexibility, which provides them with the opportunity to withstand an expanded range of environmental challenges^52^. This physiological plasticity inherent within a species can confer resilience to climate change^52^,^53^ and determine species distributions^54^. For example, flexibility in carbon acquisition can increase resilience in bleached corals^55^ and photosynthetic performances may explain depth distribution patterns of corals^56^. The envelope of responses on the physiological and developmental time scales appears to be an important determinant of adaptive performance^54^. Consequently, we suggest that the reproductive patterns exhibited by the *Alveopora* species in the MCEs of the northern GOA/E play a key role in shaping their demographic pattern, by allowing them to successfully occupy a habitat that maximizes their fitness.

This study is the first to demonstrate the reproductive cycles of two abundant MCE coral species in the northern GOA/E, reinforcing the assertion, which associates, in part, coral reproductive traits with environmental factors. Further studies of these important ecosystems are vital in order to acquire a greater understanding of the role of MCEs in the future of coral reefs.

## Methods

### Study area, sampling procedures and histological processes

The study site is located at the northern tip of the GOA/E, in front of the Interuniversity Institute for Marine Sciences in Eilat, Israel (IUI) (29°30′N, 034°55′E), at a depth of 60 m. It is a well-developed reef with a wide variety of coral species. Once a month, during full moon, nubbins (4–5 cm in length) were randomly sampled from 4–6 colonies of *Alveopora allingi* and *Alveopora ocellata* using advanced technical diving techniques (Trimix SCUBA diving). The sampled colonies were over 20 meters distant from one another. Sampling of *A. allingi* started in October 2010 and lasted until December 2011, and of *A. ocellata* it began in January 2011 and ended in February 2012. For both species two additional collections were made in September 2012, the suspected reproductive season as inferred from the histological sections. To avoid less fecund edges^57^,^58^ the nubbins were sampled from the interior rather than the circumferential part of mature (i.e. >20 cm in diameter) and healthy colonies^32^. The collected nubbins were placed in a sealed nylon bag filled with seawater and immediately after collection were fixed in 4% formaldehyde solution in seawater for 24–48 hours. Thereafter, the nubbins were rinsed in tap water for 15 minutes and transferred to 70% ethyl alcohol. The decalcification process was carried out using a combination of two chemicals: tri-sodium citrate and formic acid; a solution of 20% tri-sodium citrate in distilled water (DW) was mixed with a solution of 50% formic acid in DW at a ratio of 1:1^57^. A piece of tissue was taken from each decalcified nubbin and 6 μm thick latitudinal histological serial sections were prepared and dyed with Mayer’s hematoxylin and Putt’s eosin (H&E stain) to highlight the reproductive structures.

### Annual gonad development and fecundity

A detailed study of gonad structure, size, and development was conducted by analyzing the histological cross-sections (i.e., a total of 72 colonies of *A. allingi* and 66 colonies of *A. ocellata*). The examination included size measurements of oocytes (longest axes), performed only when the nucleoli were present, and identification of the developmental stages of gametocytes within the polyps. Mean oocyte size of each developmental stage was calculated from randomly selected oocytes. The percentage per colony of each gamete stage was calculated per sampling date, using the total number of gametes in a sample. The criteria for classification of gametocytes into developmental stages for *A. allingi* and *A. ocellata* followed that of Szmant-Froelich *et al.*^25^,^26^ and Glynn *et al.*^27^ (Supplement Table 1). The histological analysis was made using a Nikon eclipse 90i microscope and NIS Elements D 3.2 software (Nikon Instruments Inc.).

Coral fecundity was estimated by counting the number of measurable oocytes (i.e. oocytes featuring a nucleolus in the histological cross-section) per cm^2^ of a given sample on the cross-section (i.e. area of the nubbins sampled from the colonies as it appears on the slides). Fecundity estimates were measured in both species throughout the entire reproductive season.

### Temperature measurements and analysis

HOBO^®^ Pendant temperature loggers (Onset Computer Corporation) were deployed at 50 m and 10 m depth for a period of three years (September 2010 to October 2013). Temperature measurements were taken every 10 minutes (Supplement Fig. 1). The relationship between sea temperature recorded during the studied reproductive season and oocyte size was determined by Pearson correlation regression analyses (Figs 2 and 4).

### Ecological photo survey

The ecological survey was carried out by photography of random quadrats in order to quantify coral cover along a depth gradient (2–60 m) (Fig. 6). The *in-situ* photos were taken at the IUI. Fixed distance and orientation from bottom were achieved through attachment of the camera to a framer (70 × 50 cm quadrangle). Sixty photos were taken along three transects of 30 m (twenty photos per transect) at each depth: 2, 40, and 60 m. Thirty photos from each depth were randomly chosen for analysis by *CPCe 4.1* software, using ‘random point sample’ method^59^ with 200 points on each photo.

### Coral survivorship under controlled light regimes

Survivorship of the two coral species was monitored under different light conditions in the outdoor running seawater aquaria at the IUI, for a period of 12 months. Colonies of *A. allingi* and *A. ocellata* were kept under shallow-water light conditions (high intensity, full spectral irradiance simulating *ca*. 3 m depth) and under blue-light filter, “Deep blue” (Lee filters, UK) simulating light conditions at *ca.* 40 m depth (see experimental setup in Supplement Fig. 3). Survival/mortality rates were recorded every one to three months and analyzed by using the Kaplan-Meier^60^ method (Fig. 7).

### Downwelling irradiance measurements

Annual spectral downwelling irradiance measurements of λ (Ed(λ)) wavelength (Supplement Fig. 2) were conducted monthly at the IUI, using a profiling reflectance radiometer PRR800 (Biospherical Instruments, USA) which measures 19 channels (at 300–900 nm) and the integrated PAR. The PRR800 was deployed at midday (11:00–13:00), using the free-fall technique in order to avoid shade or reflectance from the boat and to maintain the light sensor in a vertical posture. The data were analyzed using the PROFILER software (Biospherical Instruments, USA).

## Additional Information

**How to cite this article**: Eyal-Shaham, L. *et al.* Reproduction, abundance and survivorship of two *Alveopora* spp. in the mesophotic reefs of Eilat, Red Sea. *Sci. Rep.* **6**, 20964; doi: 10.1038/srep20964 (2016).

## Supplementary Material

Supplementary Information

## Figures and Tables

**Table 1:** Main reproductive traits of seven coral species of the genus *Alveopora*.

	Habitat	Sexuality	Mode of reproduction	Mature oocyte size (μm)	Planulae size (μm)	Oogenesis duration (months)	Spermatogenesis duration (months)
1*Alveopora allingi*	Mesophotic reef (60m)	Hermaphrodite	Broadcast-spawning	393	–	8	3
1*Alveopora ocellata*	Mesophotic reef (60m)	Hermaphrodite	Broadcast-spawning	320	–	10	3
2*Alveopora japonica*	Shallow reef	Hermaphrodite	Brooding	787	740	11	6
3*Alveopora daedalea*	Shallow reef	Hermaphrodite	Brooding	450	700	2	1
4*Alveopora gigas*	Shallow reef	Hermaphrodite	Broadcast-spawning	–	–	–	–
4*Alveopora verrilliana*	Shallow reef	Hermaphrodite	Broadcast-spawning	–	–	–	–
4*Alveopora tizardi*	Shallow reef	Hermaphrodite	Broadcast-spawning	***–***	***–***	***–***	***–***

^1^This study.

^2^Harri *et al.*^61^.

^3^Shlesinger *et al.*^49^.

^4^Reviewed by Baird *et al.*^5^.
