# Supplementary material for: Reproduction, abundance and survivorship of two Alveopora spp. in the mesophotic reefs of Eilat, Red Sea
Source: Sci Rep. 2016 Feb 10;6:20964. doi: 10.1038/srep20964 (PMC4748239; doi:10.1038/srep20964)
Supplement: Supplementary Information [file srep20964-s1.pdf]

Reproduction, abundance and survivorship of two *Alveopora* spp. in the mesophotic reefs of Eilat, Red Sea

Lee Eyal-Shaham[1,2,#], Gal Eyal[1,2,#], Raz Tamir[2,3] and Yossi Loya[1,*]

Supplementary information:

Temperatures at shallow vs. mesophotic reefs

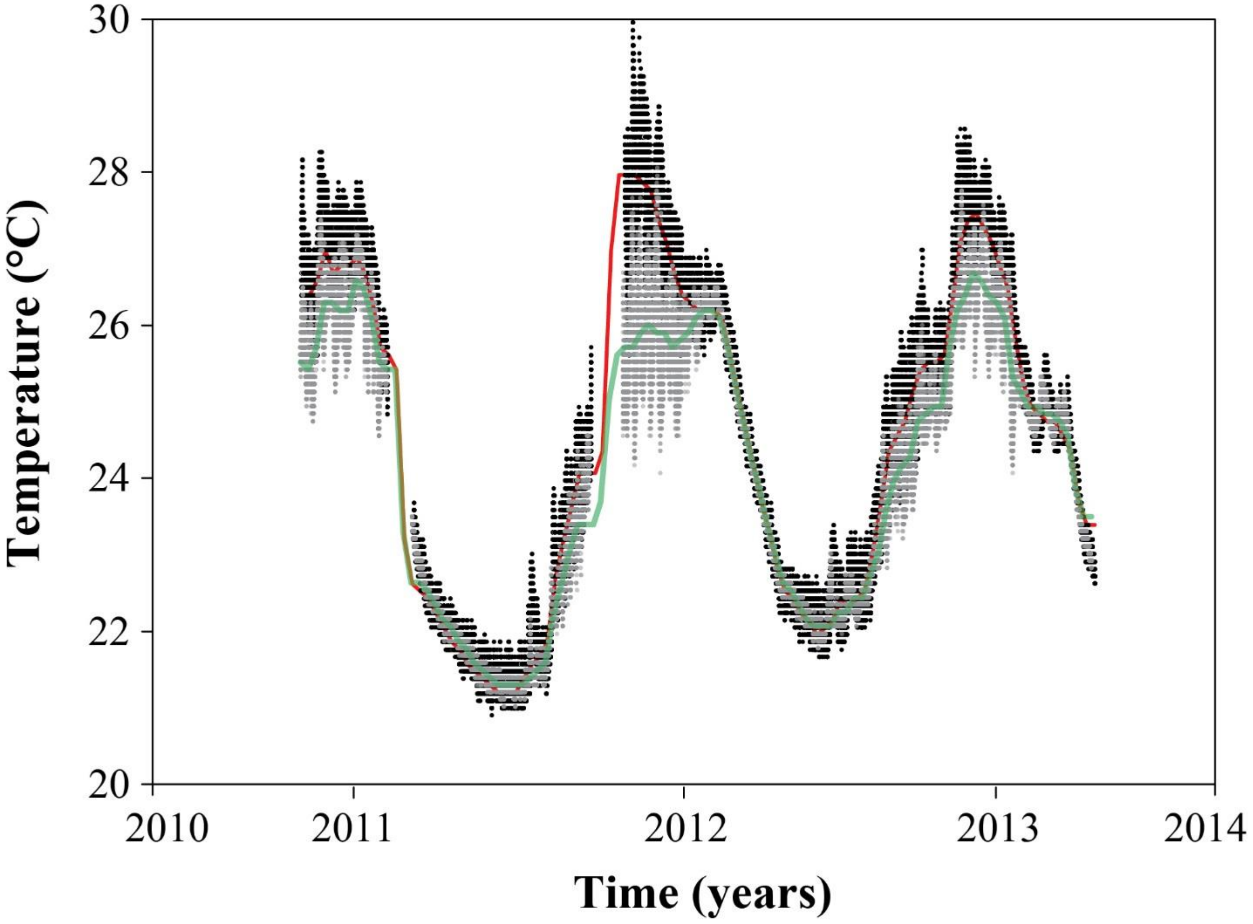

Figure 1: Temperature oscillations at shallow (10 m depth; black dotes) and mesophotic (50 m depth; grey dotes) reef zones off the IUI during three years. Temperature running medians of shallow water in red line and of mesophotic water in green. Data measurements are average of three Hobo pendent (Onset) loggers at each depth with sampling frequency of every 10 min and accuracy of 0.5°C. Missing data in 2011 and 2012 are due to periods with no data logging *in-situ*.

Changes in PAR along a depth gradient

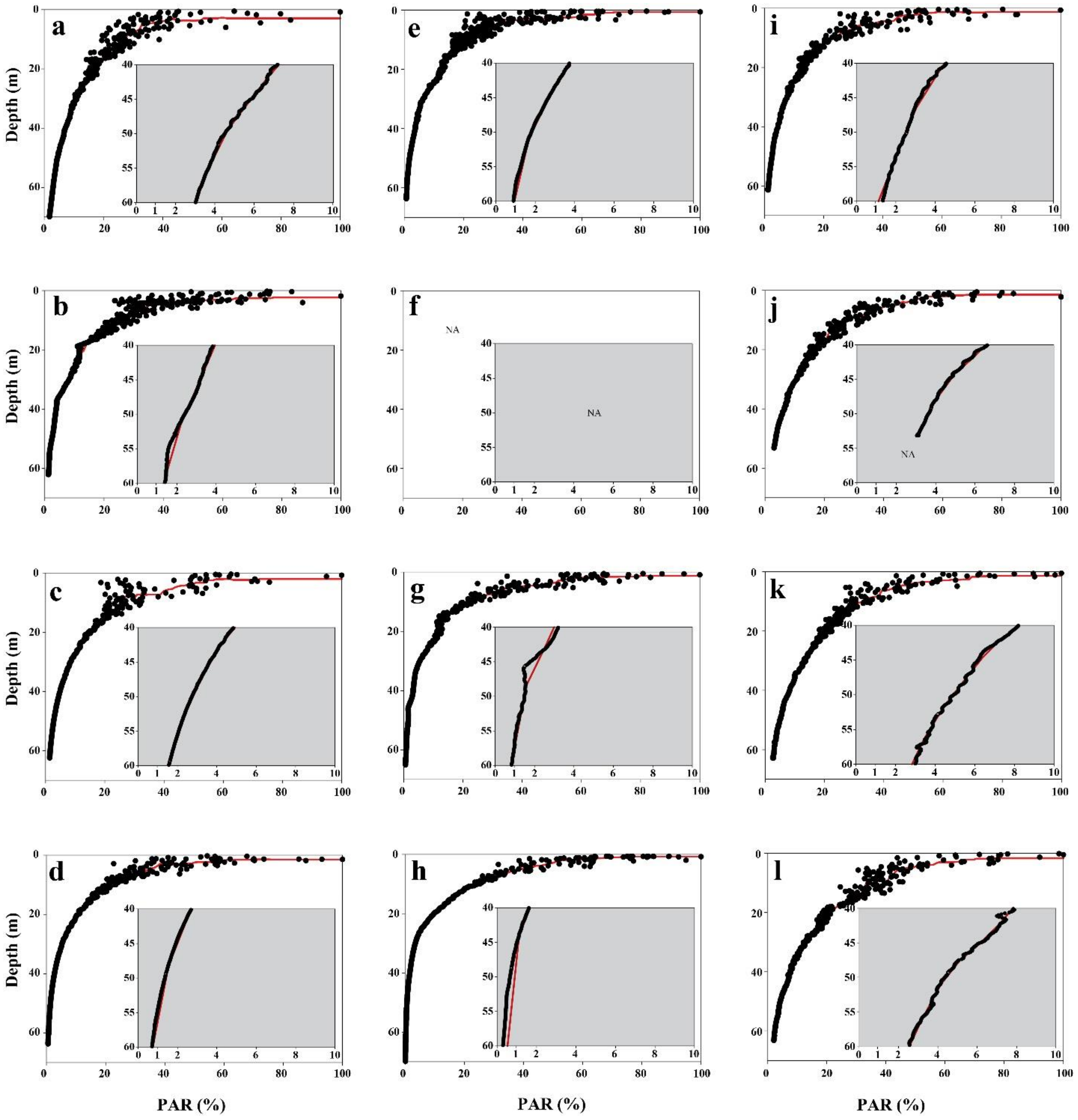

Figure 2: Changes in percentage of PAR from surface irradiance along a depth gradient offshore the IUI. Measurements where done once a month throughout the year (August 2014 - July 2015) at midday. Black dotes represent the percentage of PAR and red line represents the running median of the PAR. The inset shows the values of measurements in the *Alveopora* spp. zone (40-60m). (a) August, (b) September, (c) October, (d) November, (e) December, (f) January (no data), (g) February, (h) March, (i) April, (j) May, (k) June and (l) July.

***Ex-situ* survival experimental set-up**

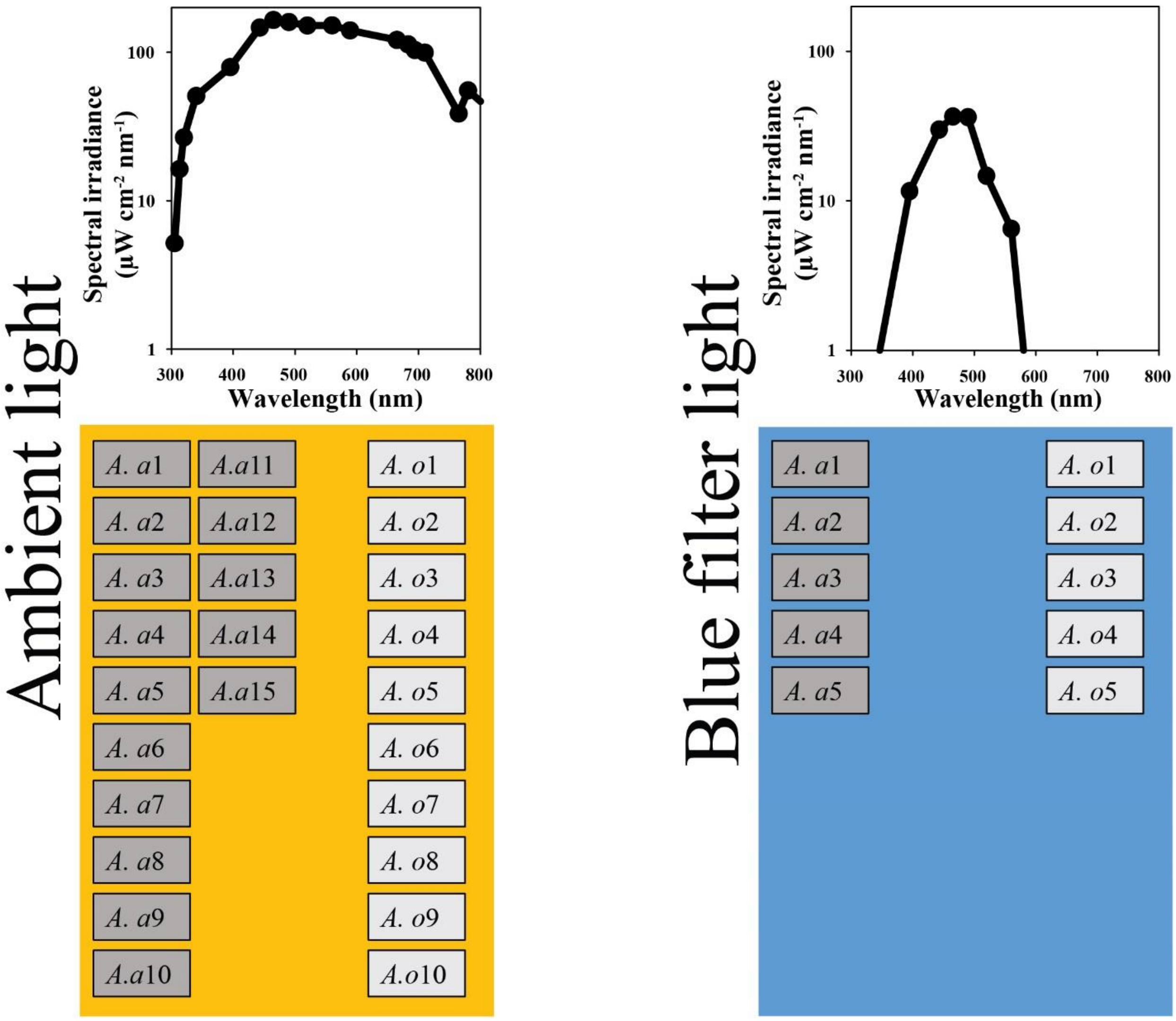

Figure 3: Experimental set-up of the manipulative monitoring of the survival rate of *Alveopora allingi* (grey aquaria; *A. a*) and *A. ocellata* (white aquaria; *A. o*) under ambient light which simulates the light at 3 m depth (orange tank) and under blue light filter which simulates the light at 40 m depth (blue tank). The set-up was placed in the running seawater system at the IUI, for a period of 12 months. Each colony was placed in a separate aquarium with separated fresh sea-water supply. Top graphs illustrate the light spectrum and intensities of the treatment at midday.

Developmental stages criteria

Table 1: Criteria for classification of gametocytes into developmental stages, after Szmant-Froelich et al.[26,27] and Glynn et al.[28]

Stage	Oocytes	Spermaries
	Light purple Oogonia, Nucleoli and cytoplasm not evident	--- -
**I**	Oocyte I located in or adjacent to mesoglea with enlarged interstitial cells	Small cluster of $\leq$ 10 cells surrounded by mesoglea
**II**	Accumulation of small amount of cytoplasm, located in mesoglea	Larger cluster of $\geq$ 10 cells surrounded by mesoglea
**III**	Increased amount of cytoplasm, but no vitelline membrane	Cells closer together, beginning of central lumen development but tails not evident
**IV**	Full sized Oocyte with well distinguished vitelline membrane. Mostly detached from mesoglea	Spermatozoa undergoing meiosis, tails in lumen

Benthic survey statistics

Table 2: One way ANOVA parameters for changes in total percent cover of all corals and percent cover of *Alveopora allingi and A. ocellata* along a depth gradient (2-60 m depth) offshore the IUI.

Source of Variation	DF	SS	MS	F	P
Total cover (all corals) between depths	2	1615	808	10.827	<0.001
Alveopora allingi cover between depths	2	1669	835	43.726	<0.001
Alveopora ocellata cover between depths	2	361	180	9.065	<0.001

Table 3: ANOVA pairwise multiple comparison (Tukey Test) for changes in total percent cover of all corals and percent cover of *Alveopora allingi* and *A. ocellata* along a depth gradient (2-60 m depth) offshore the IUI. (p) Number of compared pairs, (q) Studentized range distribution and (P) Statistical P-value.

Group	Comparison	Diff of means	p	q	P
**Total cover**	2 to 40 m depths	10.278	3	5.371	<0.001
	2 to 60 m depths	9.086	3	5.791	<0.001
	40 to 60 m depths	1.192	3	0.641	0.893
***Alveopora allingi* cover**	2 to 40 m depths	10.234	3	10.571	<0.001
	2 to 60 m depths	9.375	3	11.811	<0.001
	40 to 60 m depths	0.859	3	0.913	0.795
***Alveopora ocellata* cover**	2 to 40 m depths	5.938	3	6.005	<0.001
	2 to 60 m depths	3.456	3	3.596	0.035
	40 to 60 m depths	2.481	3	3.061	0.084
